# Ghost edge detection based on HED network

**DOI:** 10.1007/s12200-022-00036-1

**Published:** 2022-08-03

**Authors:** Shengmei Zhao, Yifang Cui, Xing He, Le Wang

**Affiliations:** 1 Institute of Signal Processing and Transmission, Nanjing University of Posts and Telecommunications (NUPT), Nanjing, 210003 China; 2 Key Lab of Broadband Wireless Communication and Sensor Network Technology (NUPT), Ministry of Education, Nanjing, 210003 China

**Keywords:** Edge detection, Ghost imaging (GI), Holistically-nested neural network, Compression ratio (CR), Signal-to-noise ratio (SNR)

## Abstract

In this paper, we present an edge detection scheme based on ghost imaging (GI) with a holistically-nested neural network. The so-called holistically-nested edge detection (HED) network is adopted to combine the fully convolutional neural network (CNN) with deep supervision to learn image edges effectively. Simulated data are used to train the HED network, and the unknown object’s edge information is reconstructed from the experimental data. The experiment results show that, when the compression ratio (CR) is 12.5%, this scheme can obtain a high-quality edge information with a sub-Nyquist sampling ratio and has a better performance than those using speckle-shifting GI (SSGI), compressed ghost edge imaging (CGEI) and subpixel-shifted GI (SPSGI). Indeed, the proposed scheme can have a good signal-to-noise ratio performance even if the sub-Nyquist sampling ratio is greater than 5.45%. Since the HED network is trained by numerical simulations before the experiment, this proposed method provides a promising way for achieving edge detection with small measurement times and low time cost.

**Graphical Abstract:**

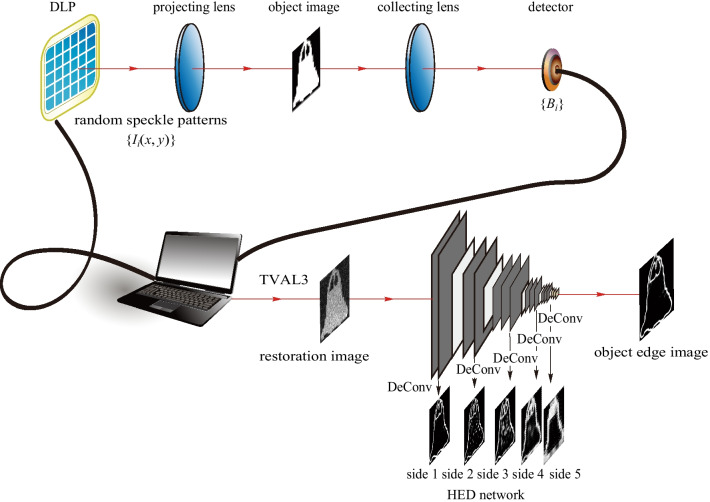

## Introduction

Ghost imaging (GI), an intriguing optical technique for imaging, is also called correlated imaging [1, 2]. Typically, in a standard GI configuration, two beams of light are presented. A signal beam is detected by a bucket detector without spatial resolution after it passes through the unknown object, while an idler beam is collected directly by a detector with spatial resolution. The image can be recovered by correlating the signal fluctuations of the signal and idler beams.

Initially, GI was experimentally demonstrated in 1995 [3] by using two-photon entanglement produced by spontaneous parametric down-conversion (SPDC). Then, GI was also found to be successfully achieved by using classical pseudothermal light sources [4], thermal source and sunlight [5, 6]. Soon, computational ghost imaging (CGI) [7] was presented to greatly simplify GI’s configuration by computing the intensity distribution of the idler beam offline, thus significantly generalized the application of GI. GI currently has a wide range of applications in laser radars [8], microscopes [9], image hiding [10], and optical encryption [11, 12], due to its robustness against hostile environments, high detection sensitivity and high spatial resolution [13–16].

In recent years, edge information detection of an unknown object based on GI was proposed [17–24] (namely ghost edge detection), where edge information could be directly detected without extracting the original image at first. For example, a gradient ghost imaging (GGI) was introduced by Liu et al*.* in Ref. [17] to directly detect the edge information of unknown objects. Subsequently, speckle-shifting GI (SSGI), an optimized edge detection scheme was proposed in Ref. [18]. Then, a similar method of subpixel-shifted GI (SPSGI) based on subpixel-shifted Walsh Hadamard speckle pattern pairs was proposed by Wang et al*.* [19], which had the advantage of improving the edge detection resolution ratio. At the same time, Yuan et al*.* [20] used structured intensity patterns to get edge information. Ren et al*.* [21] designed specific sinusoidal speckle patterns to achieve *x*-direction and *y*-direction edges of an unknown target object based on Fourier ghost imaging (FGI). In Ref. [22], a variable size Sobel operator with isotropic coefficients was devised for edge detection, which was sensitive to all directions. The compressed ghost edge imaging (CGEI) method was found to achieve the reduction in the number of measurements for edge detection by utilizing a compressive sensing technique [23]. And Ref. [24] presented a multi-directional edge detection based on gradient ghost imaging.

In recent years, deep learning (DL) had been recognized as a powerful technology for solving complex problems in computational ghost imaging [25–29]. Various network structures and training strategies based on convolutional neural networks (CNNs) have continuously improved GI performance. However, to our knowledge, there are few reports of using DL for ghost edge information detection so far.

In our paper, we present a ghost edge detection scheme based on deep learning technique, where the holistically-nested neural network is used. The so-called holistically-nested edge detection (HED) scheme combines fully CNNs with deep supervision to learn image edges effectively, and to address the challenging ambiguity problem in edge detection. The experimental data with sub-Nyquist sampling ratio from a GI system is input to the HED network, and the edge of the image is obtained from the network. The experiment results demonstrate the effectiveness of this scheme. By comparing the results with those using SSGI, CGEI and SPSGI, we verify the enhanced performance of the proposed ghost edge detection scheme.

The structure of our paper is as follows. We present the proposed ghost edge detection scheme based on HED network in Sect. 2, followed by the relevant theoretical derivations. Then, in Sect. 3, we set up a GI system and analyze the performance of the proposed ghost edge detection scheme. In Sect. 4, we present some conclusions.

## Ghost edge detection based on HED network

**Fig. 1:**
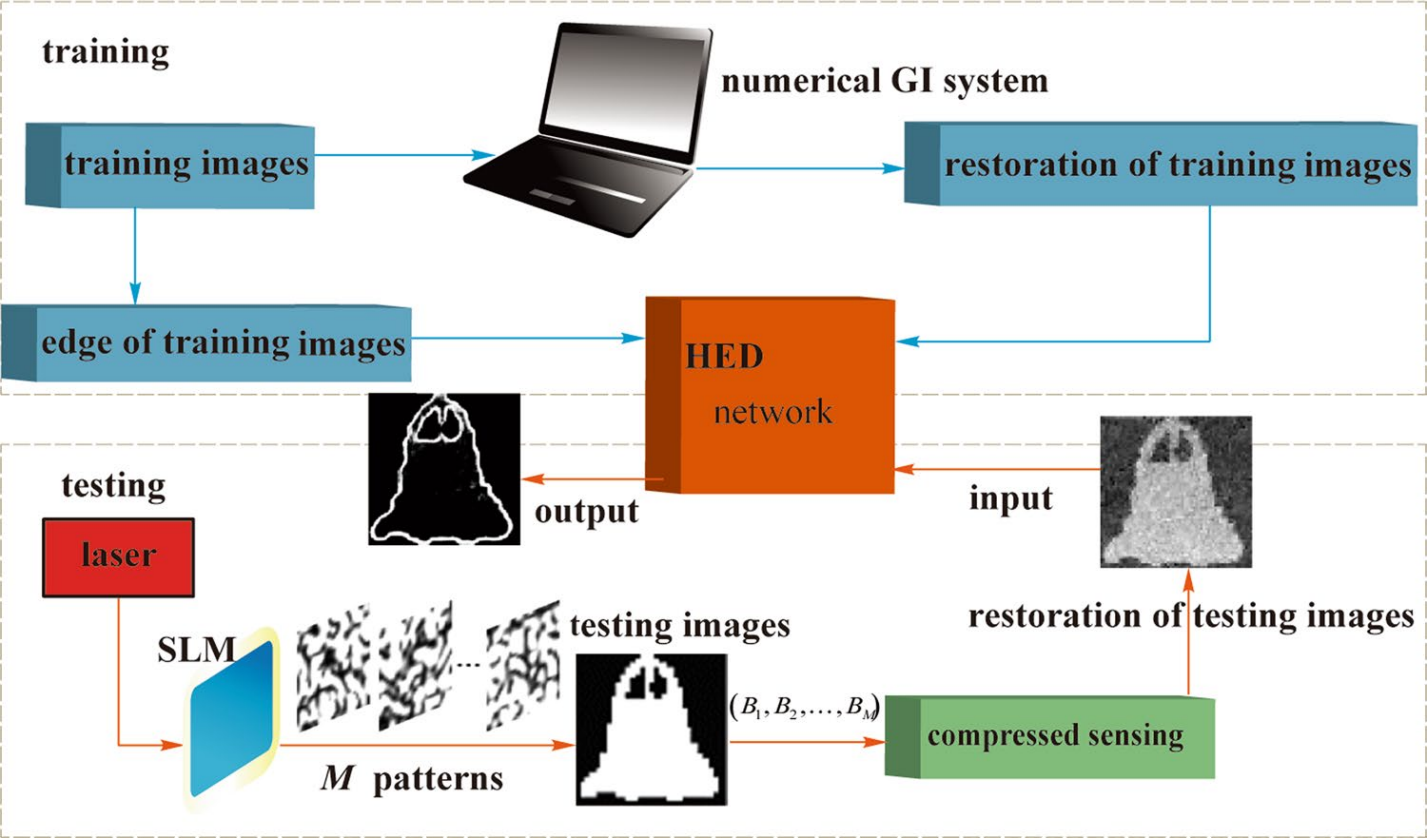
Schematic diagram of the proposed ghost edge detection scheme with deep learning

Figure 1 illustrates the schematic diagram of this proposed ghost edge detection scheme with deep learning, where the upper part is for training, and the lower part is for testing. In the training part, the train images, which come from the MINST handwritten digit database, are sampled and reconstructed by a numerical CGI system. The GI-reconstructed imaging and the edge information of the corresponding training images are used as the input and the target of the HED network, respectively, and the network’s output is the extracted edge information. In the testing part, the special speckle patterns are generated and used to illuminate the testing image, whose intensity distribution are denoted by *I*_1_(*x,y*), *I*_2_(*x,y*),…*,I*_*M*_(*x,y*), where *M* is the number of the special speckle patterns. The detected signals from the bucket detector denoted by (*B*_1_,*B*_2_,…,*B*_*M*_) can be obtained from the GI experimental system. Subsequently, the blurring of the testing image can be realized by using a compressed sensing technique with a low compression ratio (CR). When the reconstructed blurred testing image is input to the trained HED network, the clear edge information of the unknown image is obtained from the HED network.

In the experimental GI system, the detected signal *B*_*i*_ obtained in the bucket detector can be expressed as

1 $$B_{i} = \sum\limits_{x} {\sum\limits_{y} {I_{i} (x,y)T(x,y)} } ,$$

where *I*_*i*_(*x,y*) represents the *i*th special speckle pattern and random speckle patterns are used in the experiment, and *i* = 1, 2,…, *M,* *x* = 1, 2,…, *N*_*x*_, *y* = 1, 2,…, *N*_*y*_, where *N* = *N*_*x*_ × *N*_*y*_ is the pixel number of the test image. *T*(*x,y*) denotes the testing image, which can be unknown in the experiment.

The detected signals *B*_1_,*B*_2_,…,*B*_*M*_, can be rewritten as a vector ***B*** = [*B*_1_*,B*_2_,…*,B*_*M*_]^T^. And Eq. (1) can then be reformulated in a matrix form as

2 $${\varvec{B}} = {\varvec{AT}},$$

where ***A*** is a *M* × *N* matrix, whose *i*th row is the *i*th speckle pattern reshaped into a row vector with $N_{x} \times N_{y}$ elements. That is, ${\varvec{A}}_{i} = [I_{11}^{i} ,I_{12}^{i} ,...,I_{{1N_{y} }}^{i} ,I_{21}^{i} ,...,I_{{N_{x} 1}}^{i} ,...,I_{{N_{x} N_{y} }}^{i} ]$, $I_{xy}^{i} = I_{i} (x,y)$,

***T*** is a *N* × 1 column vector obtained by reshaping the two dimensional test image *T*(*x,y*). By multiplying ***A*** and ***T***, a *M* × 1 column vector is obtained, which is composed of *M* detected signals $\{ B_{i} \}_{i = 1}^{M}$.

Notice that the majority of natural images can be in sparse representation when an appropriate basis is selected, such as in the discrete wavelet transform (DWT), discrete cosine transform (DCT). Assume that the testing image *T*(*x,y*) is *K-*sparse in the DWT basis Ψ. Since matrix ***A*** used in the GI experiment is an independent and random matrix, it satisfies the restricted isometry property (RIP) [30]. Therefore, ***T*** can be obtained by solving Eq. (2) using a compressed sensing (CS) recovery algorithm.

Here, the total variation augmented Lagrangian and alternating direction algorithm (TVAL3) [31, 32] is employed. Therefore, the objective function for the testing image is

3 $$\min \sum\limits_{K} {\left\| {D_{k} {\varvec{T}}} \right\|_{1} + \frac{\mu }{2}} \left\| {{\varvec{B}} - {\varvec{AT}}} \right\|_{2}^{2} ,$$

where *D*_*k*_***T*** is the discrete gradient at component *k*; *k* = 1,2,…*,N*; *µ* is a nonnegative coefficient used to balance the regularization and fidelity of the data, and is set to 2^12^ in this work; ∥ · ∥_1_ and ∥ · ∥_2_ indicate the *l*_1_ norm and *l*_2_ norm, respectively. The TVAL3 algorithm is chosen since it can solve non-smooth and unconstrained optimization problems, which exists widely in image processing applications. TVAL3 is superior to other widely used algorithms in speed and quality of reconstruction.

**Fig. 2:**
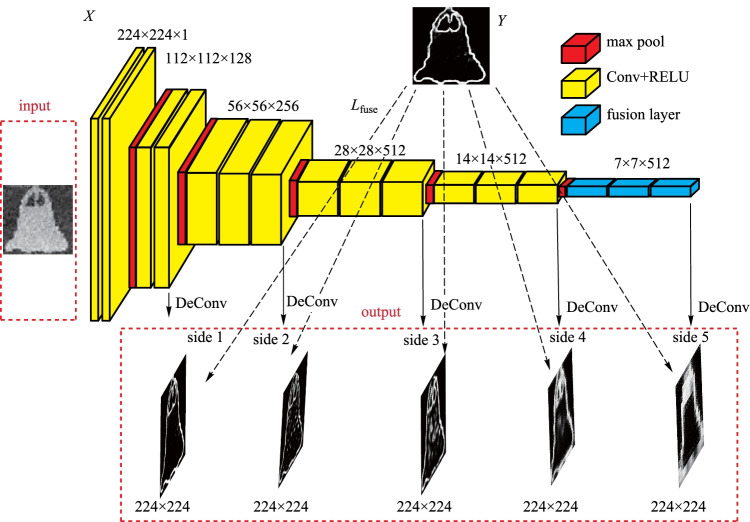
Structure of the holistically-nested edge detection network

Figure 2 shows the HED network structure. Prediction from image to image can be carried out by HED by utilizing the deeply-supervised nets and fully convolutional neural networks [33–35]. HED network can automatically learn rich hierarchical representations (under the guidance of deep supervision of side responses), which are beneficial for dealing with the ambiguity problem that is challenging in the detection of image edges.

The training set for HED is denoted by *S* = {(*X*_*n*_,Y_*n*_)}, *n* = 1,2,…*,Q*, where $X_{n} = \{ x_{j}^{(n)} \}$ stands for the original input image, such as the input MINST images, and $Y_{n} = \{ y_{j}^{(n)} ,y_{j}^{(n)} \in \{ 0,1\} \}$ stands for binary edge information of the ground truth corresponding to image *X*_*n*_; *j* = 1,2,…, ∥*X*_*n*_∥, ∥*X*_*n*_∥ = *N* represents the number of pixels in an image.

Supposing that the network has all the network layer parameter sets ***W***, and *P* side output layers, which are linked to *P* classifiers, and the relevant weights are expressed as ***w*** = (***w***^(1)^,..., ***w***^(*P*)^). The HED’s objective function is given by

4 $$L_{{{\text{side}}}} ({\varvec{W}},{\varvec{w}}) = \sum\limits_{m = 1}^{P} {\alpha_{m} \ell_{{{\text{side}}}}^{(m)} ({\varvec{W}},{\varvec{w}}^{(m)} )} ,$$

where $\ell_{\text{side}}^{(m)}$ (***W***,***w***^(*m*)^) denotes the loss function of the *m*th side output layer; *α*_*m*_ denotes the weight in the summation; The distribution of edge/non-edge pixels is significantly biased for typical natural images; therefore, a balanced class of cross-entropy loss function is considered [33, 35],

5 $$\ell_{{{\text{side}}}}^{(m)} ({\varvec{W}},{\varvec{w}}^{(m)} ) = - \beta \sum\limits_{{j \in Y_{ + } }} {\log \Pr (y_{j} = 1|X;{\varvec{W}},{\varvec{w}}^{(m)} )} - (1 - \beta )\sum\limits_{{j \in Y_{ - } }} {\log \Pr (y_{j} = 0|X;{\varvec{W}},{\varvec{w}}^{(m)} )} ,$$

where *β* = ∥*Y*_−_∥*/*∥*Y* ∥, 1 − *β* = ∥*Y*_+_∥*/*∥*Y* ∥, ∥*Y*_−_∥ and ∥*Y*_+_∥ stand for the edge and non-edge ground truth label sets, respectively. $X_n = \{ x_{j}^{(n)} \}$ is one training image; $Y_{n} = \{ y_{j}^{(n)} ,y_{j}^{(n)} \in \{ 0,1\} \}$ is its edge map, Furthermore, $\hat{Y}_{{{\text{side}}}}^{(m)} = \Pr (y_{j} = 1|X;{\varvec{W}},{\varvec{w}}^{(m)} ) = \sigma (\alpha_{j}^{(m)} )$ denotes the predicted edge value at the *j*th pixel of the *m*th output layer, where *σ*(·) ∈ [0,1] represents the Sigmoid function. The fusion layer is represented by the weighted summation of the *m*th side output layers, that is

6 $$\hat{Y}_{{{\text{fuse}}}} = \sigma \left[ {\sum\limits_{m = 1}^{P} {h_{m} \hat{A}_{{{\text{side}}}}^{(m)} } } \right],$$

where *h*_*m*_ denotes the weighting coefficient, $\hat{A}_\text{side}^{(m)} = \{ \alpha_{j}^{(m)} ,j = 1, \ldots ,\left\| X \right\|\}$ are the activations of the *m*th layer side output, $\alpha_{j}^{(m)}$ is the activation value at pixel *j* of the *m*th side output layer. The cross-entropy loss function is adopted for the fusion layer, *L*_fuse_(***W***, ***w***,* h*) = Dist(*Y,*$\hat{Y}$_fuse_), where Dist(·,·) is the distance between the fused predictions and the ground truth label.

Finally, the objective function is

7 $${\text{argmin}}\left( {L_{{{\text{side}}}} \left( {{\varvec{W}},{\varvec{w}}} \right) \, + L_{{{\text{fuse}}}} \left( {{\varvec{W}},{\varvec{w}},h} \right)} \right),$$

where ***W***, ***w***, *h* are the variables to be optimized.

In testing, the output of weighted-fusion layer and the side output layers can be predicted by the input image as

8 $$(\hat{Y}_\text{fuse} ,\hat{Y}_\text{side}^{(1)} , \ldots ,\hat{Y}_\text{side}^{(P)} ) = {\text{NN}}(X,({\varvec{W}},{\varvec{w}},h)^*),$$

where NN (·) represents the network generated edge graphs, ()* means the optimum value. The output of HED is the mean of fusion layer and side output layers as

9 $$\hat{Y}_{{{\text{HED}}}} = {\text{average}}(\hat{Y}_\text{fuse} ,\hat{Y}_\text{side}^{(1)} , \ldots ,\hat{Y}_\text{side}^{(P)} ).$$

In our HED network, there are 5 side output layers. The size of the input image is 224 × 224 × 1. For simplification, the fusion layer is represented by the weighted first side output layer. All the mapped edge outputs from the 5 side layers are 224 × 224 pixels. In the first side output layer, the size of the convolution kernel and stride of the first side layer are 1 and 2 × 2, respectively. From the second side layer, the size of the convolution kernel and stride of the latter side layer is twice that of its the previous layer. The loss function for each side output layers is optimized to make the output edge image approach the true edge. Additionally, we subtract the mean value from the reconstructed image before it is input to the HED network, so that the loss function can converge more smoothly.

We use the signal-to-noise ratio (SNR) to quantitatively estimate the edge detection performance, which is defined as [19, 21, 22]

10 $${\text{SNR = }}\frac{{{\text{mean}}(T_{{{\text{edge}}}} ) - {\text{mean}}(T_{{{\text{back}}}} )}}{{({\text{var}} (T_{{{\text{back}}}} ))^{0.5} }},$$

where *T*_back_ and *T*_edge_ are the background grayscale values and edge grayscale values of the edge detection result, respectively. var(·) denotes the variance value, and mean(·) represents the mean value. Generally, the quality of edge detection is directly proportional to the SNR. Meanwhile, the definition of compression ratio (CR) is introduced as

11 $${\text{CR = }}\frac{M}{{N_{x} \times N_{y} }},$$

where *M* is the number of measurement; *N*_*x*_ and *N*_*y*_ denote the horizontal and vertical dimensions of the testing image, *N* = *N*_*x*_ × *N*_*y*_.

## Experimental results and discussion

In this part, we verify the proposed ghost edge detection scheme by experiments based on CGI configuration. The numerical computations are implemented at Dell Optiplex 920 with Intel Core i7-4790 CPU and 24 GB memory and 3.6 GHz intel core. All 3000 handwritten digit images are chosen from the MNIST database. For each handwritten image, we train the HED 10 times, and the handwritten digit (28 × 28 pixels) is enlarged to 224 × 224 pixels by padding before it is input to the HED network. The epoch of the training is set to 300. The batch size is 10. The weight decay coefficient is 0.0002. And the initial weight of each layer is 1.

**Fig. 3:**
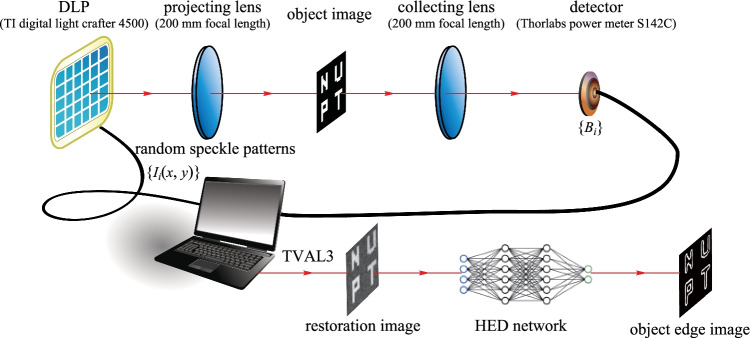
Experimental setup of the ghost edge detection scheme based on HED network. DLP: digital light projector

The experiment is carried out according to Fig. 3. The random speckle patterns are generated by the computer and are then loaded to a digital light projector (DLP), TI Digital light Crafter 4500. The random speckle light beam is expanded through a 200 mm projector lens and illuminate the test image (64 × 64 pixels). Finally, we can obtain the detected signal *B*_*i*_ from a bucket detector (Thorlabs power meter S142C) using a collecting lens with 200 mm focal length. With the TVAL3 method, the blurring images are reconstructed from a few detected signals and the corresponding random speckle patterns. Since the HED has already been trained by 3000 handwritten digit images, the clear edge information can be extracted after the blurred image is enlarged and input to the trained HED network.

**Fig. 4:**
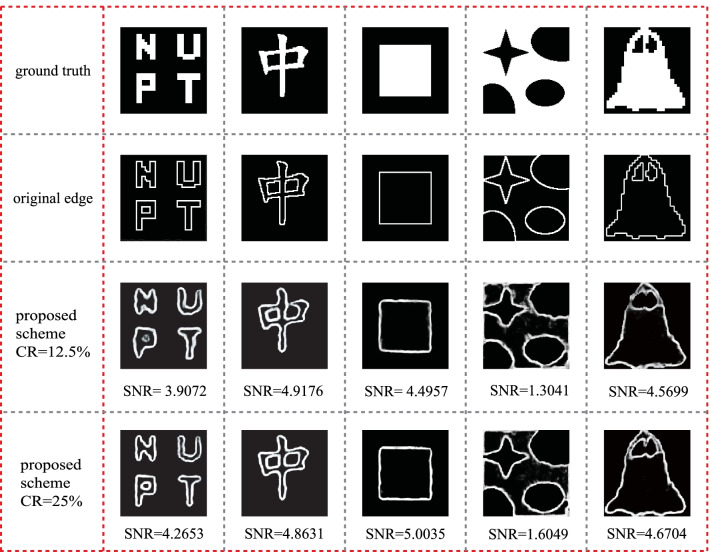
Experimental results with the proposed scheme, where “original edges” are achieved by applying Sobel operator on the “ground truth”

The experimental results of this proposed ghost edge detection method are shown in Fig. 4. The results with CR = 12.5% and CR = 25% are presented. Different CRs are achieved by adjusting the effective number of measurements, that is, the size of *M*. It is shown that the edges extracted by using the proposed ghost edge detection scheme are almost identical to those original edges when the CR is 25%. For a lower CR of 12.5%, one can also get a reasonable edge information. At the CR of 12.5%, it takes about 16.17 s to generate GI image through the TVAL3 algorithm. And it takes about 4.9 s to obtain an edge detection result through the trained HED network.

**Fig. 5:**
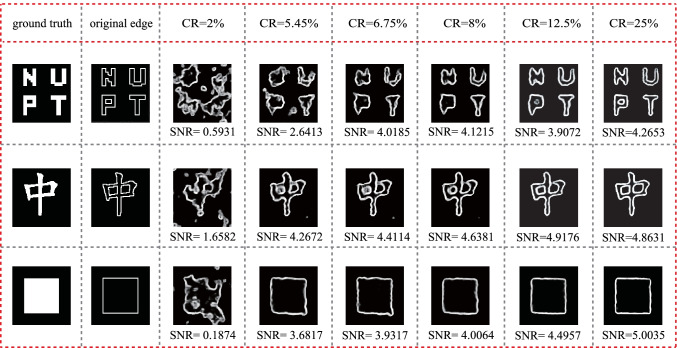
Experimental results obtained by using proposed ghost edge detection scheme with different CRs

Figure 5 shows the experimental results obtained by using the proposed ghost edge detection scheme for lower CRs, where CR is set in the range from 2% to 25%. Experimental results indicate that our proposed scheme provides an increasing quality of edge information when CR increases. Normally, this proposed scheme has a good performance when CR ≥ 8%, and the SNR changes a little when CR ≥ 12.5%. The results demonstrate that this method can provide high quality edge information with a sub-Nyquist sampling ratio.

**Fig. 6:**
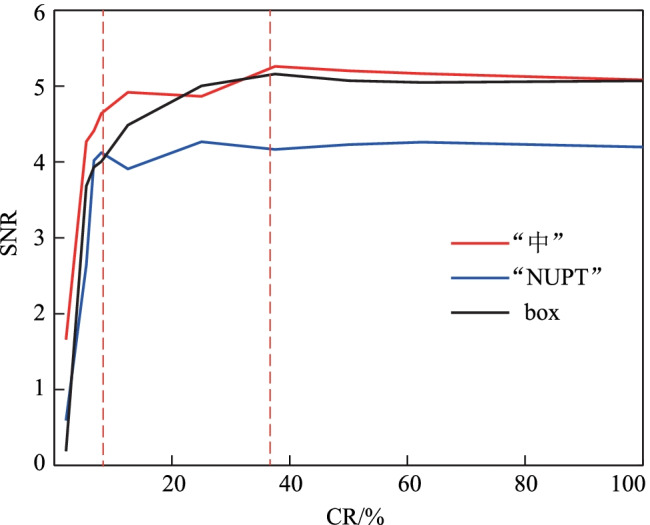
SNR performance versus CR for different target objects

To further verify the performance of this proposed ghost edge detection scheme, we present the SNRs of the obtained edge image for testing images with different CRs in Fig. 6. The results show that the SNRs for the reconstructed edges quickly increase with the increased CR at sampling ratios lower than sub-Nyquist sampling ratio. Figure 6 presents the SNR performance of edge detection within the full range of CR for different target images. It is found that the SNR has an abrupt increase when CR ≤ 8% and then increases slowly with CR in the range of 8% ≤ CR ≤ 37.5%. When CR ≥ 37.5%, the SNR of the edge image is almost unchanged. It is further suggested that the proposed ghost edge detection scheme can have a better performance with a sub-Nyquist sampling ratio.

**Fig. 7:**
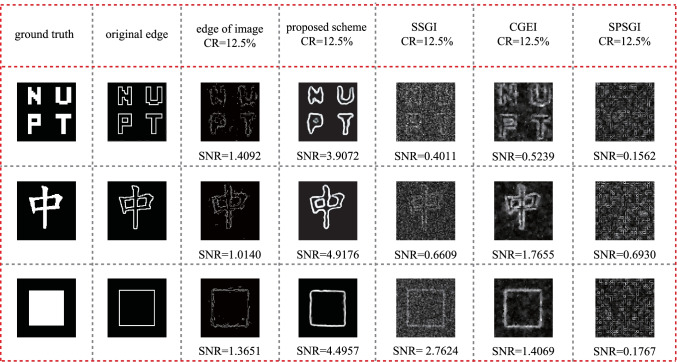
Comparison of the proposed scheme with those results by using SSGI scheme, CGEI scheme, SPSGI scheme with random speckle patterns, together with edge of image when CR = 12.5%

Lastly, we present SNR performance comparison of the edge detection scheme proposed in this paper with SSGI, CGEI and SPSGI in Fig. 7, where “original edge” is obtained by applying Sobel operators on the “ground truth”; the edge of the constructed image is obtained by applying Sobel operators on the reconstructed image from GI experiment system. Here, SPSGI is the subpixel-shifted edge detection scheme with random speckle patterns [19]. The results show that, compared with those of SSGI, CGEI, and SPSGI scheme, as well as the edge of the constructed image, the proposed scheme has a better SNR performance. For the “NUPT” image, the SNR value by using the proposed method is 3.9072, while the SNR values are 0.4011 by using SSGI, 0.5239 by using CGEI, 0.1562 by using SPSGI and 1.4092 for the edge of constructed image. The SNR performance is improved by 874%, 646%, 2401% and 177.3% respectively. For the “box” image, the SNR value by using our method is 4.4957, while the SNR values are 2.7624 by using SSGI, 1.4069 by using CGEI, 0.1767 by using SPSGI and 1.3651 for the edge of the constructed image. Therefore, the SNR performance is improved by 62.7%, 219.5%, 2244.2%, 229.3% respectively. Our scheme has a better SNR performance compared with other methods due to HED being based on the ideas of deeply supervised nets and fully convolutional neural networks.

Notice that all schemes use random speckle patterns, and the CRs are all set as 12.5% for comparison. The worse performance of SPSGI is caused by the usage of random speckle pattern instead of the Walsh Hadamard speckle pattern.

## Conclusions

This paper presents a ghost edge detection scheme based on an HED network, where an HED network has been trained with simulation data and the edge information of unknown objects has been obtained by experimental data from a GI system. The experimental results indicate that the proposed ghost edge detection scheme can extract edge information with high quality even if the CR of the image is low. Compared with SSGI, CGEI and SPSGI edge detection schemes, the proposed scheme exhibits an improvement in SNR. In addition, the HED network in the proposed scheme can be trained by images obtained from CGI system before experiment, thus the time to achieve the edges from the experimental data is reduced.
